# The association of zonulin-related proteins with prevalent and incident inflammatory bowel disease

**DOI:** 10.1186/s12876-021-02075-y

**Published:** 2022-01-03

**Authors:** Xiao Wang, Ashfaque A. Memon, Karolina Palmér, Anna Hedelius, Jan Sundquist, Kristina Sundquist

**Affiliations:** 1 Department of Clinical Sciences Malmö, Center for Primary Health Care Research, Institutionen För Kliniska Vetenskaper, Malmö (IKVM), Lund University, Inga-Marie Nilssons gata 53, Wallenberg Laboratory, plan 5, Box 50332, 202 13 Malmö, Sweden; 2 Department of Family Medicine and Community Health, Department of Population Health Science and Policy, Icahn School of Medicine at Mount Sinai, New York, NY USA; 3 Center for Community-Based Healthcare Research and Education (CoHRE), Department of Functional Pathology, School of Medicine, Shimane University, Matsue, Japan

**Keywords:** Inflammatory bowel disease, Crohn’s disease, Ulcerative colitis, Zonulin, Zonulin-related proteins (ZRP)

## Abstract

**Background:**

Current evidence regarding the association of serum zonulin-related proteins (ZRP) levels with prevalent inflammatory bowel disease (IBD) is contradictory. Moreover, the association with the subsequent risk of incident IBD is still unexplored. This study aimed to investigate the association of serum ZRP levels with both prevalent and incident IBD.

**Method:**

The study included a total of 130 women (51–61 years) from the Women’s Health in Lund Area (WHILA) study, which included 18 prevalent IBD (diagnosed before baseline) and 47 incident IBD diagnosed during the 17 years (median) follow-up and age- and sampling time-matched controls. Serum ZRP was tested in all participants by ELISA.

**Results:**

The serum ZRP levels were significantly higher in prevalent IBD compared to their matched controls (63.2 ng/ml vs 57.0 ng/ml, *p* = 0.02), however, no evidence of a difference in ZRP levels was found between the women who developed IBD during the follow-up period and their matched controls (61.2 ng/ml vs 59.7 ng/ml, *p* = 0.34). Using linear mixed models, we found that the association between serum ZRP levels and prevalent IBD (β = 6.2, *p* = 0.01), remained after adjusting for potential confounders. Conditional logistic regression models showed no evidence of an association between ZRP level and incident IBD (OR 1.03, *p* = 0.34).

**Conclusion:**

Higher serum ZRP levels were associated with prevalent IBD, but not with incident IBD in our study samples.

**Supplementary Information:**

The online version contains supplementary material available at 10.1186/s12876-021-02075-y.

## Background

Inflammatory bowel disease (IBD), including Crohn’s disease (CD) and ulcerative colitis (UC), is characterised by chronic inflammation of the gastrointestinal (GI) tract. UC is an inflammation of the colon and rectum, whereas CD is characterised by mucosal inflammation in different areas of the GI tract [1]. Diarrhea and bleeding are more common in UC, while in CD abdominal pain and weight loss are more common. CD is predominantly observed among women in adulthood, whereas UC is equally observed in both sexes [2]. IBD is a chronic disease that is very common in the population with a prevalence range of 200–400 cases per 100,000 individuals in Western countries [3–6]. The pathogenesis of IBD is still unclear; the current hypothesis is that IBD results from an abnormal immune response to the commensal microbiota in the gut in genetically susceptible populations [4]. The intestinal barrier dysfunction may increase permeability, which allows for the interaction between exterior antigens and mucosal immune system, and then lead to a dysregulated response [7]. Animal models showed that increased intestinal permeability could proceed with the development and relapse of IBD [8–10]. There is one reported case of CD development predicted by an abnormal permeability test in healthy women with a positive family history of CD [11]. Therefore, intestinal permeability may be crucial in IBD, but whether it is a cause or consequence of IBD is still under-investigated.

Zonulin was discovered and first described by Fasano approximately 20 years ago and it can increase permeability in the epithelial layer of the small intestine and contributes to intestinal innate immunity [12, 13]. Recently, it was demonstrated that zonulin is a group of structurally and functionally related proteins whose origin is the precursor of protein haptoglobin 2 (pre-HP2) [14]. Therefore, it is considered to be more appropriate to use the term zonulin-related proteins (ZRP) rather than the term zonulin [15–17]. Therefore, the term ZRP will be applied in the present study. Circulating plasma/serum ZRP has been suggested as a potential marker of intestinal permeability [18–20]. Increased serum/plasma ZRP levels have been associated with a wide range of chronic diseases, such as celiac disease [12, 21], type 1 and 2 diabetes [22], and mental disorders [23]. However, the results are contradictory and no definite conclusions have been drawn, there is insufficient information about ZRP 's role in some important states of IBD [17]. The role of ZRP in the onset of IBD remains not fully understood.

In the present study, we aimed to investigate the potential association of serum ZRP levels with the prevalence and incidence of IBD in selected samples from a prospective study followed for 20 years.

## Material and methods

### Study population

**Fig. 1:**
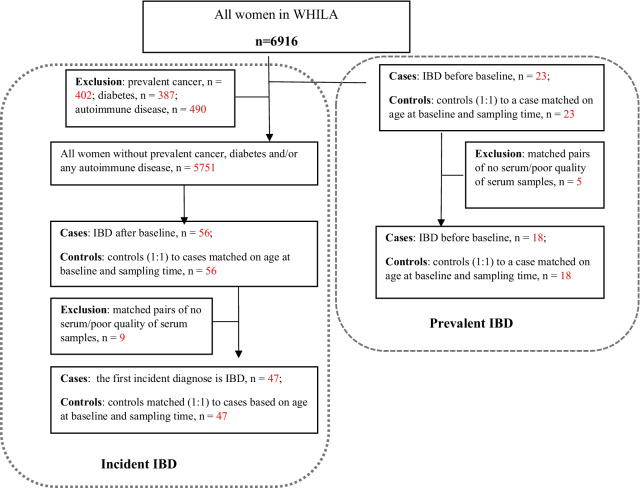
Flowchart of the study populations

The study population was selected from the Women’s Health in Lund Area (WHILA) study. All women living in Lund, Sweden between 1995 and 2000, which were born between 1935 and 1945 (n = 10,766), were invited to a health survey. In the WHILA study, a total of 6917 women (64.2%) completed the questionnaire and underwent a physical examination [24]. In brief, all the participants received the questionnaire and answered it within two hours after written consent. Participants were followed-up until death, or if no event occurred until May 31st, 2015. There was no financial reimbursement for participation. In the present study, we selected all the patients who were diagnosed with IBD by using the International Classification of Diseases (ICD) codes as following: 555, 556, K50, and K51. Prevalent cases were those which were diagnosed before baseline. Incident cases were diagnosed during the follow-up period. The flow chart of the study population was shown in Fig. 1. In the whole study population (n = 6916), a total of 23 participants were diagnosed with IBD before baseline. Twenty-three non-IBD participants at baseline were matched on age and sampling time (1:1). We further excluded participants who have been diagnosed with cancers, diabetes and autoimmune diseases as these diseases were reported to be associated with ZRP levels [25, 26], and finally, a total of 56 participants were diagnosed with IBD during the follow-up period. Fifty-six participants without IBD at baseline and during follow-up were selected and matched with IBD cases conditional on age and sampling time (1:1). A total of 158 serum samples were selected for ZRP testing, out of which 28 samples were excluded because of the poor quality/small quantity serum (Fig. 1).

### Blood sampling

The whole blood was collected into serum separate tubes (SST) and centrifuged at 2000 g for 10 min. Serum was harvested and stored at − 80 °C until the time of measurement.

### ZRP measurement

The serum ZRP concentration was measured by competitive ELISA method (Immundiagnostik AG, Bensheim, Germany) according to the manufacturer's instructions. In brief, 20 ul serum was used for testing and optical density was read at 450 nm. The 4-parameter algorithm was used to set up the standard curve. The final ZRP concentration (ng/ml) was calculated based on the standard curve. The inter-assay coefficient of variance (CV) was 5.5% and intra-CV was 3.6%.

### Statistical analysis

Data were presented as the mean and standard deviation (SD) together with min and max for age, body mass index (BMI), systolic blood pressure (SBP), diastolic blood pressure (DBP), Triglycerides (TG), total cholesterol (TC), high-density lipoprotein cholesterol (HDL-C) and low-density lipoprotein cholesterol (LDL-C), whereas smoking and alcohol use were presented as numbers and percentages. ZRP levels were presented with mean and SD, median and IQR together with min and max.

To examine the association between ZRP levels and subject characteristics we used linear mixed models with ZRP levels as outcome variables to take into account the correlation within matched pairs.

Paired t-test and Wilcoxon signed-rank test were used to compare the ZRP levels between cases and controls, for all women as well as stratified by UC and CD (Additional file 1: Table S1).

We used regression models to be able to adjust for potential confounders. For the incident population, we used a conditional logistic regression model with case versus control as outcome, estimating odds ratios (OR). For the prevalent population, we used a linear mixed model with ZRP levels as outcome, estimating regression coefficients (β). We adjusted for BMI, smoking condition and alcohol use because these variables have been shown to be associated with both IBD and ZRP levels previously [27–31]. STATA version 16 (StataCorp LP) was used for all statistical analyses.

### Ethical permission

All the participants provided written consent according to the Declaration of Helsinki before enrollment in the study. The study was approved by the Ethics Committee in Lund, (2011/494 and 2015/6).

## Results

### Characteristics of the study population according to serum ZRP levels

A total of 130 women, 65 cases (18 prevalent IBD and 47 developed IBD during follow-up) and 65 matched controls were included in the analysis. The mean age of the study population was 55 years and the mean BMI was 24.6 at baseline. Most participants were non-smokers (78%) and low consumers of alcohol (68%). The mean serum ZRP level was 60.4 ng/ml (Table 1). No big differences were seen between cases and controls in either the prevalent or incident population.

**Table 1:** Study population characteristics at baseline

Variable	Incident		Prevalent		All
	Cases (n = 47)	Controls (n = 47)	Cases (n = 18)	Controls (n = 18)	All (n = 130)
*Age*					
Mean (SD)	55.7 (2.9)	55.9 (2.8)	54.2 (2.3)	54.6 (2.2)	55.4 (2.8)
Min–max	52–61	52–61	51–60	52–60	51–61
Number non-missing	47	47	18	18	130
*BMI*					
Mean (SD)	24.7 (3.3)	24.7 (3.3)	24.3 (4.3)	24.3 (3.2)	24.6 (3.4)
Min–max	19.7–38.4	18–33	16.8–33.1	18.9–32	16.8–38.4
Number non-missing	44	42	17	17	120
*Smoking condition*					
Yes or former/no (N)	12/34	12/35	1/17	4/14	29/100
%	26/74	26/74	6/94	22/78	22/78
Number non-missing	46	47	18	18	129
*Alcohol use*					
Grams per day, 0/0.1–11.9 /> = 12 (N)	4/32/10	8/33/5	6/10/1	5/11/1	23/86/17
%	9/70/22	17/72/11	35/59/6	29/65/6	18/68/13
Number non-missing	46	46	17	17	126
*Systolic blood pressure (SBP)*					
Mean (SD)	132 (17.1)	126 (13.5)	129 (19.8)	130 (17.3)	129.1 (16.3)
Min–max	105–165	100–157	95–180	110–165	95–180
Number non-missing	47	47	18	18	130
*Diastolic blood pressure (DBP)*					
Mean (SD)	84.6 (10.5)	81.9 (8.6)	84.9 (9.9)	82.9 (8.1)	83.5 (9.4)
Min–max	70–110	67–100	60–105	70–100	60–110
Number non-missing	47	47	18	18	130
*TG (Triglycerides)*					
Mean (SD)	1.8 (0.9)	1.5 (0.7)	1.6 (0.8)	1.6 (1.0)	1.7 (0.8)
Min–max	0.7–4.3	0.6–3.9	0.7–3.5	0.6–5.2	0.6–5.2
Number non-missing	47	47	18	18	130
*TC (Total cholesterol)*					
Mean (SD)	5.8 (1.2)	5.9 (1.0)	5.9 (1.2)	6.0 (1.2)	5.9 (1.1)
Min–max	3.2–8.4	3.5–8.3	4.1–8.2	4.4–8.4	3.2–8.4
Number non-missing	47	47	18	18	130
*HDL-C (High-Density Lipoprotein Cholesterol)*					
Mean (SD)	1.7 (0.4)	1.7 (0.4)	1.8 (0.4)	1.8 (0.4)	1.7 (0.4)
Min–max	0.8–2.6	1.0–2.6	1.3–2.6	1.1–2.4	0.8–2.6
Number non-missing	47	47	18	18	130
*LDL-C (Low-Density Lipoprotein Cholesterol)*					
Mean (SD)	3.4 (1.1)	3.3 (0.7)	3.3 (1.1)	3.4 (1.2)	3.4 (1.0)
Min–max	1.2–6.3	1.7–4.9	2.0–5.7	1.9–5.9	1.1–6.3
Number non-missing	44	43	16	17	120
*Glucose*					
Mean (SD)	6.1 (1.0)	6.0 (0.9)	6.4 (2.8)	5.9 (1.1)	6.1 (1.4)
Min–max	4.4–9.2	3.6–9.3	4.5–17	4.2–8.7	3.6–17
Number non-missing	47	47	18	18	130
ZRP *(ng/ml)*					
Mean (SD)	61.2 (9.9)	59.7 (10.7)	63.2 (8.5)	57.0 (9.7)	60.4 (10.0)
Median (IQR)	62.2 (14.5)	61.3 (15.8)	63.7 (14.6)	58.3 (14.7)	61.5 (14.8)
Min–max	39.7–81.1	40.6–80.8	51.1–78.5	40.9–72.8	39.7–83.1
Number non-missing	47	47	18	18	130

BMI, Body Mass Index; SD, standard deviation; IQR, Interquartile Range

We further investigated the association between ZRP levels and metabolic risk markers. Linear mixed models of age, body mass index (BMI), systolic blood pressure (SBP), diastolic blood pressure (DBP), Triglycerides (TG), total cholesterol (TC), high-density lipoprotein cholesterol (HDL-C), low-density lipoprotein cholesterol (LDL-C), smoking condition, and alcohol use on serum ZRP levels indicated that ZRP levels were higher in subjects with a higher BMI (β = 0.6, *p* = 0.02), higher DBP (β = 0.2, *p* = 0.03), higher TG (β = 2.1, *p* = 0.048), higher LDL-C (β = 2.2, *p* = 0.009) and a lower HDL-C (β = − 5.7, *p* = 0.002) (Table 2).

**Table 2:** Association between ZRP levels (ng/ml) and subject characteristics at baseline (n = 130)

Variable	Number of non-missing	Regression coefficient	*P* value^a^	95% CI
Age	130	0.5	0.17	− 0.2; 1.2
BMI	120	0.6	0.02	0.1; 1.0
Smoking condition (ref: no) Yes or former	129	− 2.6	0.19	− 6.5; 1.3
Alcohol abuse (ref: 0.1–11.9 g)				
0 g		− 0.5	0.82	− 5.0; 4.0
> = 12	126	− 4.8	0.05	− 9.6; 0.05
Systolic blood pressure (SBP)	130	0.08	0.12	− 0.02; 0.2
Diastolic blood pressure (DBP)	130	0.2	0.03	0.02; 0.3
TG	130	2.1	0.048	0.02; 4.1
TC	130	1.0	0.16	− 0.4; 2.5
HDL-C	130	− 5.7	0.002	− 9.2; − 2.1
LDL-C	120	2.2	0.009	0.6; 3.8
Previous IBD (ref: no) Yes	130	4.7	0.04	0.3; 9.0
Future IBD (ref: no) Yes	130	1.4	0.36	− 1.6; 4.4

SBP, Systolic blood pressure; DBP, Diastolic blood pressure; TC, Total cholesterol; TG, Triglycerides; HDL-C, High-density lipoprotein cholesterol; LDL-C, Low-density lipoprotein cholesterol

^a^Association tested by linear mixed models with ZRP asthe outcome

### IBD and serum ZRP levels

Using a paired t-test, we found that serum ZRP levels were significantly higher in prevalent cases of IBD compared to controls, the mean level was 63.2 ng/ml and 57.0 ng/ml, respectively (*p* = 0.02), but there was no significant difference in ZRP levels between incident cases of IBD and controls (61.2 vs. 59.7, *p* = 0.34) (Table 3). This was also examined by using the Wilcoxon signed-rank test and the results remained unchanged. When stratifying for UC and CD, we also found higher ZRP levels in prevalent cases compared to controls (Additional file 1: Table S1). To investigate the association between prevalent IBD and ZRP levels, while adjusting for possible confounders, we performed a linear mixed model analysis. Our results showed that serum ZRP levels were associated with the prevalent IBD (β = 6.2, *p* = 0.01) and that the results remained the same after adjusting for BMI, smoking condition, and alcohol (Table 4). To test the association between ZRP levels and the risk of IBD, we applied conditional logistic regression and we found no evidence of an effect on IBD susceptibility (OR 1.03, *p* = 0.34) (Table 4).

**Table 3:** ZRP levels (ng/ml) in relation to various disease conditions

Variable	Incident			Prevalent		
	ZRP cases (n = 47)	ZRP controls (n = 47)	*P* value	ZRP cases (n = 18)	ZRP controls (n = 18)	*P* value
*All*						
Mean (SD)	61.2 (9.9)	59.7 (10.7)	0.34^a^	63.2 (8.5)	57.0 (9.7)	0.02^a^
Median (IQR)	62.2 (14.5)	61.3 (15.8)	0.45^b^	63.7 (14.6)	58.3 (14.7)	0.03^b^
Min–max	39.7–81.1	40.6–80.8		51.1–78.5	40.9–72.8	
Number non-missing	47	47		18	18	

^a^Difference between cases and controls tested by paired t-test (means)

^b^Wilcoxon signed-rank test (medians)

**Table 4:** Adjusted analysis of the association between ZRP levels (ng/ml) and disease conditions

	Incident (n = 94)		Prevalent (n = 36)	
	OR^a^ (95% CI)	*P* value^b^	β^c^ (95% CI)	*P* value^d^
Univariate	1.03 (0.97; 1.08)	0.34	6.2 (1.3; 11.2)	0.01
*Adjusted for:*				
BMI	1.03 (0.97; 1.10)	0.32	6.3 (1.0; 11.7)	0.02
Smoking	1.03 (0.98; 1.09)	0.26	5.7 (0.4; 11.0)	0.04
Alcohol	1.03 (0.97; 1.09)	0.28	7.4 (2.8; 12.1)	0.002

^a^Odds for having IBD versus not for a unit increase in ZRP

^b^Association tested by a conditional logistic regression model

^c^Difference in ZRP at baseline between previous IBD versus no previous IBD

^d^Association tested by linear mixed model

## Discussion

By utilising a prospective study, WHILA biobank samples, we observed that prevalent IBD cases had higher serum ZRP levels compared to matched controls, whereas there was no association between serum ZRP levels and incident IBD. Moreover, we found that ZRP levels were associated with metabolic risk markers, BMI, DBP, TG, LDL-C and HDL-C.

ZRP is an analogue of a cholera toxin that is capable of regulating intestinal permeability and was first described by Fasano approximately 20 years ago [32]. During the past two decades, ZRP has been described as a serum biomarker of intestinal permeability in different diseases in preclinical and clinical studies [17]. The underlying pathogenesis of IBD is still unclear, although increased intestinal permeability has been shown to be attributed to the pathogenesis of IBD. However, the exact mechanism that leads to the increase of intestinal permeability in IBD is still under investigation. To date, there are only two clinical studies that have investigated the association between serum ZRP levels and IBD. In agreement with our results, Caviglia et al. [33] reported higher serum ZRP levels in IBD patients compared to healthy controls. Another study by Malickova found that serum /fecal ZRP levels were higher only in Crohn’s disease but not in ulcerative colitis [34]. ZRP upregulation has been detected in both the acute phase and after anti-inflammatory treatment of IBD [25]. Under the inflammatory condition, gut dysbiosis may induce epithelial cells to produce an increased amount of ZRP to the intestinal lumen/the blood circulation and subsequently lead to abnormal intestinal barrier function, followed by exterior antigens to enter the bloodstream and then trigger the excess immune response which in turn leads to further leakiness [35]. Together, this may explain our finding that serum ZRP levels were higher in prevalent IBD cases compared to controls.

However, none of the published studies have explored the potential association of ZRP levels with incident IBD. To our knowledge, this is the first clinical study to investigate the role of serum ZRP as a predictor biomarker in IBD. Our finding, regarding the association with incident IBD, suggests that ZRP may not be a predictor for IBD incidence. Most of the incident IBDs were diagnosed more than two years after the baseline (blood sample), thus indicating that we may exclude the possibility that ZRP levels might predict short-term incident IBD.

Previous studies reported that serum ZRP was related to metabolic risk markers, such as BMI, DBP, TG, LDL-C, and HDL-C [18, 27, 36–39]. In the present study, we also confirmed previous findings on the associations between ZRP and these risk factors.

The main limitation in the current study was the study sample size being relatively small and calls for further research with a larger population. Nonetheless, to our knowledge, this is the first clinical study to investigate serum ZRP as a predictor for IBD. This could be the starting point for further studies to examine the clinical role of serum ZRP in IBD. In addition, it is necessary to mention another important limitation; the serum measurements were based on a commercially available ELISA kit [16, 23]. Recent studies raised important concerns regarding the specificity of the measurement of zonulin. It is likely that the kit detects other unknown members of the zonulin family rather than only zonulin [40, 41]. Therefore, the findings are needed to be validated by an improved ELISA kit with the development of specific and reliable monoclonal antibodies for zonulin/pre-HP2.

In conclusion, we found an association of serum ZRP levels with prevalent IBD, but not with incident IBD in our study population. Therefore, the impaired intestinal permeability may be a consequence, rather than a cause of the chronic inflammatory response that characterizes both CD and UC. The results need further study in a large cohort to confirm.

## Supplementary Information

**Additional file 1**.** Table S1**. Zonulin levels in relation to various disease conditions.

## Data Availability

The clinical data for the present study will not be shared publicly as participants were informed at the time of providing consent that only researchers involved in the project would have access to the information they provided. Please contact the corresponding author for more information.

## Abbreviations

IBD: Inflammatory bowel disease
CD: Crohn’s disease
UC: Ulcerative colitis
WHILA: Women’s Health in Lund Area
pre-HP2: Protein haptoglobin 2
ZRP: Zonulin-related proteins
SBP: Systolic blood pressure
DBP: Diastolic blood pressure
TG: Triglycerides
TC: Total cholesterol
HDL-C: High-density lipoprotein cholesterol
LDL-C: Low-density lipoprotein cholesterol
SD: Standard deviation
BMI: Body mass index
CI: Confidence interval
IQR: Interquartile range
