# Supplementary material for: The association of zonulin-related proteins with prevalent and incident inflammatory bowel disease
Source: BMC Gastroenterol. 2022 Jan 3;22:3. doi: 10.1186/s12876-021-02075-y (PMC8725386; doi:10.1186/s12876-021-02075-y)
Supplement: Supplementary file 1 — Additional file 1. Table S1. Zonulin levels in relation to various disease conditions. [file 12876_2021_2075_MOESM1_ESM.docx]

| **Table S1 Zonulin levels in relation to various disease conditions** | | | | | | |
| --- | --- | --- | --- | --- | --- | --- |
|  | Incident | | | Prevalent | | |
| Variable | Zonulin cases  (n = 47) | Zonulin controls  (n = 47) | P-value^a^ | Zonulin cases  (n = 18) | Zonulin controls  (n = 18) | P-value^a^ |
| *Ulcerative colitis*  Mean (SD)  Median (IQR)  Min-max  Number non-missing | 62.3 (10.4)  63.9 (14.8)  39.7-80.5  31 | 62.1 (10.9)  65.2 (17.9)  40.6-80.8  31 | 0.89 | 63.1 (8.2)  63.2 (13.6)  51.1-78.5  11 | 57.8 (10.3)  60.6 (16.0)  40.9-72.8  11 | 0.15 |
| *Crohn's disease*  Mean (SD)  Median (IQR)  Min-max  Number non-missing | 59.1 (8.9)  59.1 (9.8)  46.1-83.1  16 | 55.1 (8.9)  55.2 (15.3)  40.6-68.2  16 | 0.12 | 63.4 (9.5)  67.8 (18.5)  51.3-73.9  7 | 55.9 (9.4)  57.3 (14.4)  45.0-71.4  7 | 0.06 |

^a^Difference between cases and controls tested by Wilcoxon signed-rank test

|  |
| --- |
|  |
